# The interplay between glutamatergic circuits and oxytocin neurons in the hypothalamus and its relevance to neurodevelopmental disorders

**DOI:** 10.1111/jne.13061

**Published:** 2021-11-16

**Authors:** Amanda B. Leithead, Jeffrey G. Tasker, Hala Harony‐Nicolas

**Affiliations:** ^1^ Department of Psychiatry Icahn School of Medicine at Mount Sinai New York NY USA; ^2^ Seaver Autism Center for Research and Treatment New York NY USA; ^3^ Department of Neuroscience Icahn School of Medicine at Mount Sinai New York NY USA; ^4^ Friedman Brain Institute at the Icahn School of Medicine at Mount Sinai New York NY USA; ^5^ Neurobiology Division Department of Cell and Molecular Biology Tulane University New Orleans LA USA; ^6^ Mindich Child Health and Development Institute at the Icahn School of Medicine at Mount Sinai New York NY USA

**Keywords:** glutamate, hypothalamus, lactation, neurodevelopmental disorders, oxytocin

## Abstract

Oxytocin (OXT) neurons of the hypothalamus are at the center of several physiological functions, including milk ejection, uterus contraction, and maternal and social behavior. In lactating females, OXT neurons show a pattern of burst firing and inter‐neuron synchronization during suckling that leads to pulsatile release of surges of OXT into the bloodstream to stimulate milk ejection. This pattern of firing and population synchronization may be facilitated in part by hypothalamic glutamatergic circuits, as has been observed in vitro using brain slices obtained from male rats and neonates. However, it remains unknown how hypothalamic glutamatergic circuits influence OXT cell activity outside the context of lactation. In this review, we summarize the in vivo and in vitro studies that describe the synchronized burst firing pattern of OXT neurons and the implication of hypothalamic glutamate in this pattern of firing. We also make note of the few studies that have traced glutamatergic afferents to the hypothalamic paraventricular and supraoptic nuclei. Finally, we discuss the genetic findings implicating several glutamatergic genes in neurodevelopmental disorders, including autism spectrum disorder, thus underscoring the need for future studies to investigate the impact of these mutations on hypothalamic glutamatergic circuits and the OXT system.

## Graphical abstract

Oxytocin (OXT) neurons of the hypothalamus are involved in several physiological functions, including milk ejection, uterus contraction, and maternal and social behavior. Synchronized, burst firing of oxytocin neurons in the hypothalamus during lactation leads to pulsatile release of oxytocin into the bloodstream to stimulate milk ejection. Glutamate has been shown to influence this pattern of oxytocin neural activity in both in vivo and in vitro models. Extrahypothalamic glutamatergic inputs innervate oxytocin neurons, yet their role in modulating oxytocin neural activity outside the context of lactation is not fully understood and could be of relevance for neurodevelopmental disorders (NDDs), given the recent discoveries of several NDDs‐risk genes encoding for glutamatergic synaptic components. We propose that future studies directed at dissecting the role of glutamatergic circuits in regulating the OXT system and modulating social behaviors will enhance our understanding of the mechanisms of function of the OXT system, and that investigating the impact of NDDs‐associated mutation on the OXT system has the potential to uncover new pathophysiology that could be underlying social behavior deficits.

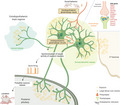

## 1 INTRODUCTION

Oxytocin (OXT) neurons of the hypothalamus are at the center of several physiological functions, including behavior.^1^, ^2^ These neurons are classified as magnocellular and parvocellular neurons, mainly based on their shape, size and intranuclear localization. They also differ in their projection targets and implications in distinct brain circuits.^3^, ^4^, ^5^, ^6^, ^7^, ^8^, ^9^, ^10^, ^11^ Magnocellular OXT neurons are located in three hypothalamic nuclei: the paraventricular nucleus (PVN), the supraoptic nucleus (SON) and the accessory nuclei, whereas parvocellular OXT neurons are mainly located in the PVN.^7^ Early immunohistochemical studies in mammalian species have demonstrated that axonal projections of OXT neurons are spread throughout the central nervous system and that magnocellular and parvocellular neurons each have a specific pattern of distribution.^6^ Magnocellular OXT neurons project to the posterior pituitary where OXT is released into the circulation to regulate peripheral functions, including milk ejection and parturition, a pathway that does not implicate parvocellular OXT neurons.^7^, ^12^ Parvocellular neurons, in contrast, project to the midbrain, brain stem and spinal cord^8^, ^13^ to modulate cardiovascular function, breathing, feeding behavior and nociception.^14^, ^15^, ^16^, ^17^, ^18^ Through their collateral axons, magnocellular neurons of both the PVN and SON also project to other brain targets, including the prefrontal cortex, anterior olfactory nucleus, lateral septum, medial and central amygdala, and nucleus accumbens, to modulate a variety of behaviors, including social behavior.^4^, ^5^, ^6^, ^19^, ^20^, ^21^ Less is clear, however, about how somatosensory and social cues are transmitted to OXT neurons and which neural inputs play a role in regulating the activity of OXT neurons to modulate physiological functions, including behavior.

In this review, we discuss research highlighting the influence of glutamate on OXT neural activity in vivo during lactation and parturition in females and in vitro using hypothalamic slices primarily obtained from adult males and neonates. We also emphasize the need for future studies to investigate whether hypothalamic glutamatergic circuits contribute to physiological functions other than parturition and lactation in females and/or males in vivo. Furthermore, we speculate that investigating the impact of mutations in glutamatergic genes on these circuits, as well as on the OXT system, will be of significant relevance to neurodevelopmental disorders characterized by social behavior deficits, including autism spectrum disorder (ASD).

## 2 ELECTROPHYSIOLOGICAL CHARACTERISTICS OF OXYTOCIN NEURONS IN VIVO

Much of our knowledge about the characteristics of OXT neurons is gleaned from in vivo electrophysiological experiments conducted in the 1970s and 1980s, which were performed on anesthetized lactating female rats.^12^ These studies were pioneered by Wakerley and Lincoln, who characterized the activity of antidromically‐identified neurosecretory cells in the PVN and SON during lactation.^22^, ^23^, ^24^ They found that the majority of these neurosecretory cells engage in consistent, low frequency activity (“tonic” firing) (1–10 spikes s^–1^), whereas a small percentage of these cells display intermittent activity separated by periods of quiescence, which they described as a “phasic” pattern of discharge. Furthermore, they reported that 10–20 s prior to increases in intramammary pressure indicative of milk ejection, about half of the recorded neurosecretory cells accelerate to a high‐frequency rate of discharge (30–80 spikes s^–1^). This high‐frequency firing lasts for 2–4 s, occurs at regular intervals of 4–8 min with transient periods of inhibition, and is strongly influenced by the number of suckling pups. These findings led to the speculation that high‐frequency or “burst” firing underlies the release of pulses of OXT into the bloodstream during lactation, when OXT action on the mammary gland is needed for milk let‐down. This speculation was supported and further delineated by a subsequent study conducted in 1977 by Poulain and colleagues, who demonstrated that, in both the PVN and SON, there are at least two distinct sets of neurosecretory cells, one of which displays “burst” firing during milk ejection (OXT neurons) and the other that displays an asynchronous “phasic” pattern of firing in response to hemorrhage (vasopressin [AVP] neurons). The background firing rates of neurosecretory cells were also further differentiated as fast continuous (> 3 spikes s^–1^) or slow irregular (< 3 spikes s^–1^), the latter of which was more common.^25^ Importantly, the same firing pattern of OXT neurons prior to milk let‐down was also observed in unanesthetized rats and further recorded in the expulsive phase of parturition, indicating that OXT burst activity is indeed associated with these naturally occurring conditions.^26^, ^27^ It was later established that OXT concentration in the blood increases significantly during nursing and parturition,^28^ which aligned with the electrophysiological firing pattern of OXT neurons observed during these physiological conditions.

Following these findings, it was theorized that, for OXT to be released into the bloodstream at high levels during lactation, OXT neurons must fire bursts at the same time. To address this theory, Belin, Moos and Richard investigated the relationship of the firing patterns of multiple OXT neurons by carrying out paired extracellular recordings in bilateral SON nuclei or PVN/SON nuclei in lactating female rats.^29^, ^30^ They found that peak activation of bursts in pairs of OXT neurons occurred coincidentally, with minor differences noted in the degree of synchronization between neurons (i.e., time to burst onset, maximum firing rate and amplitude). Furthermore, they observed that, throughout milk ejection, cells could become “recruited”, such that if one cell in a pair was engaged in burst firing when the other was not, over time, the non‐bursting cell might begin to burst as well and, once it did, its peak activation was synchronized with that of the other bursting cell in the pair.^31^ They therefore concluded that burst activity of OXT neurons between nuclei is highly synchronized and thereby facilitates the pulsatile release of OXT.

## 3 ELECTROPHYSIOLOGICAL CHARACTERISTICS OF OXYTOCIN NEURONS IN VITRO

These ground‐breaking studies performed in vivo were followed by several in vitro studies, which used acute hypothalamic slices and organotypic slice cultures to further inform the mechanisms of neuronal activity. In vitro studies offer the advantage of being able to control the extracellular environment and perform more stable intracellular recordings. Furthermore, the general pattern of activity of hypothalamic neurosecretory cells in vivo has also been observed in vitro, with the majority of cells engaging in spontaneous slow irregular firing, whereas some display fast continuous or burst firing patterns.^32^, ^33^, ^34^, ^35^, ^36^ However, it is important to note that key differences in the characteristics of neurosecretory cell activity in vivo and in vitro have been reported, particularly in relation to their interspike interval (ISI) distributions.^37^ It is assumed that the loss of synaptic input caused by deafferentiation during slice preparation led to these observed differences in ISI distribution between in vivo and in vitro data. Computational analysis with a Hodgkin–Huxley model derived from in vitro data confirmed that the ISI distributions of in vitro and in vivo data could be similarly matched if the in vitro model was stimulated with tonic excitatory input.^38^ Thus, tonic excitatory synaptic input to the OXT neurons appears to play an important role in influencing specific aspects of the pattern of neurosecretory cell activity. Additionally, among studies using in vitro models, reports concerning the specific characteristics of firing patterns have varied greatly, which could be attributed to several other factors, including slice preparation and origin (adult males, lactating females, neonates), the region of recording (SON or PVN) and the type of neuron recorded (AVP or OXT).

More targeted electrophysiological recordings of hypothalamic neurosecretory cells in vitro became possible with the development of specific antibodies to identify these cell types, which could be used in conjunction with dye‐labeling of recorded neurons to differentiate between OXT and AVP neurons.^39^, ^40^ Using this approach, groups targeted their intracellular recordings specifically to the OXT neural population and again found similarities as well as differences in the firing pattern of OXT neurons between previous recordings in lactating females in vivo^12^, ^29^, ^30^, ^41^, ^42^ and in slices obtained from lactating rats, adult males or neonates in vitro.^43^, ^44^, ^45^, ^46^, ^47^, ^48^ In vitro, OXT neuron bursting activity consisted of bursts of smaller amplitude, more variable duration and shorter inter‐burst intervals compared to the typical pattern of bursting activity observed in vivo. It was also reported by Wang and Hatton, using paired extracellular recordings in vitro, that the bursting activity of OXT neurons was not synchronized in slices obtained from lactating rats as it is in vivo in lactating rats.^45^ Contrastingly, Israel and colleagues observed synchronized burst activity between pairs of OXT neurons using intracellular recordings in hypothalamic organotypic culture; however, considerable variability in the degree of burst synchronization was reported.^43^ As noted previously, these observed differences between in vitro and in vivo models are not entirely surprising because many inputs to the hypothalamus are severed in the process of slice preparation. Yet, providing proper stimulants in the media, such as phenylephrine, low Ca^2+^, OXT and glutamate,^44^, ^46^ can allow studies to parse out the relative influences of these substances and piece together the mechanism by which specific patterns of OXT neural activity occur. In this way, in vitro studies have led to critical advances in our knowledge of OXT neural activity, although limitations of the model should also be acknowledged with these findings.

## 4 POSITIVE FEEDBACK REGULATION OF OXYTOCIN NEURONS BY OXYTOCIN

The idea of using OXT to stimulate the firing of OXT neurons was formulated based on early evidence suggesting that OXT neurons are capable of regulating their own activity and the paracrine release of OXT by a positive feedback mechanism. Intracerebroventricular administration of OXT in lactating female rats was found to cause an increase in the firing frequency and in the burst amplitude and frequency of OXT neurons.^49^ These increases were accompanied by a rise in intramammary pressure and milk ejection. Furthermore, administration of an OXT receptor antagonist inhibited both bursting activity and lactation. Interestingly, i.c.v. OXT administration was also capable of eliciting bursts in previously non‐responsive neurons^29^, ^31^ and OXT injected directly into the PVN or SON induced bursting activity in the contralateral nuclei,^50^ together indicating that local OXT is important for the recruitment of hypothalamic OXT neurons involved in the milk ejection reflex. To further examine whether local endogenous OXT acts on OXT neurons, microdialysis was performed in females during parturition and lactation, which confirmed that intranuclear concentrations of OXT were indeed increased in the PVN and SON during these processes.^51^, ^52^, ^53^ Importantly, the increase in OXT was attenuated following infusion of an OXT receptor antagonist into the hypothalamus, which also negatively impacted milk ejection and parturition.^54^, ^55^ From these studies, it has been proposed that intrahypothalamic OXT, released from the soma and dendrites of OXT neurons,^56^ regulates OXT burst activity via positive feedback. Computational modeling has provided support for this theory,^57^ indicating that OXT exerts a local excitatory effect that, in conjunction with tonic afferent excitatory synaptic drive and phasic inhibitory effects of local endocannabinoid release, shapes the pattern of burst firing.

Similar mechanisms of OXT action on hypothalamic OXT neurons has also been observed in vitro. OXT administered through medium to slices of hypothalamus taken from adult male or lactating female rats led to an increase in OXT release, which could be blocked by an OXT antagonist.^58^ Extracellular recordings in hypothalamic slices of male rats further revealed that application of OXT excites putative OXT neurons, causing an increase in their firing rate.^59^ Addition of OXT or OXT receptor agonist to hypothalamic neonatal slice culture also led to increased bursting activity of both spontaneously bursting and non‐bursting hypothalamic OXT neurons, which could be inhibited by an OXT antagonist.^43^, ^48^ Interestingly, hyperpolarization/depolarization was unable to affect OXT‐induced bursting activity, whereas application of the glutamate receptor antagonist 6‐cyano‐7‐nitroquinoxaline‐2,3‐dione (CNQX) was able to do so. These results led to the conclusion that synaptic input involving glutamate, coupled with OXT, is highly influential in the coordination of burst activity in hypothalamic OXT cells. However, as previously noted, the pattern of OXT activity observed in vitro differs from that observed in vivo. In vivo, OXT application stimulates bursting but does not increase background firing rate, as it does in vitro.^29^, ^31^, ^48^, ^49^, ^50^ Furthermore, a suckling stimulus is necessary to induce bursting in vivo.^49^ Therefore, although in vitro models provide important information concerning how OXT regulates the activity of OXT neurons, the underlying synchronous bursting mechanism may be more complicated as a result of intact intrinsic and extrinsic influences in vivo.

## 5 GLUTAMATE REGULATES OXYTOCIN NEURONAL ACTIVITY IN THE HYPOTHALAMUS

### 5.1 In vivo studies

The excitatory amino acid neurotransmitter glutamate has been shown to play an important role in stimulating OXT activity within the hypothalamus, thereby regulating OXT release.^60^ In female rabbits, application of glutamate by microelectrophoresis in the PVN increased the firing rate of neurosecretory and non‐neurosecretory cells.^61^ Microinjection of l‐glutamate directly into the PVN of male rats also led to increased concentration of OXT in the blood, which was coupled with decreased heart rate.^62^ Similar results were obtained by Hattori and colleagues, showing decreased heartbeat following l‐glutamate infusion.^63^ In their study, they also employed microdialysis to measure OXT concentration in the PVN following l‐glutamate infusion, but reported no changes in central OXT levels. It is possible that the microdialysis and RIA approach was not sensitive enough to detect changes in PVN dialysate OXT, whereas changes in plasma OXT were of a high magnitude (seven‐fold increase) and could be easily detected by these methods.

In an effort to further understand the role of glutamate receptor subtypes in regulating OXT neural activity, OXT release and its physiological relevance, the effect of specific excitatory amino acid agonists and antagonists on OXT release in lactating rats was assessed. Glutamate receptors can be ionotropic (fast) or metabotropic (slow), with the former consisting of NMDA receptors and non‐NMDA receptors (AMPA and kainate).^64^ In their initial studies, Parker and Crowley found that AMPA and kainic acid injected into the SON led to increased concentration of OXT in the plasma.^65^ Furthermore, they found that the effects of AMPA were attenuated by the AMPA/kainate receptor antagonist CNQX. Interestingly, suckling‐induced increases in OXT concentration were also inhibited by CNQX, indicating that glutamate may be important for the physiological regulation of OXT release during lactation. Notably, application of NMDA or the metabotropic receptor agonist (l*S*,3*R*)‐l‐amino‐ cyclopentane‐1,3‐dicarboxylic acid had no effect. However, further investigation revealed a partial role for NMDA receptors in OXT release, given that co‐application of AMPA and NMDA agonists in the SON or PVN stimulated increases in plasma OXT that were then blocked by either AMPA or NMDA antagonists (CNQX and 3‐[2‐carboxypiperazin‐4‐yl]propyl‐1‐phosphonic acid, respectively).^66^ A separate study by Moos and colleagues later identified a role for NMDA receptors in OXT cell activity in lactating rats.^67^ Injection of NMDA or the NMDA antagonist dl‐2‐amino‐5‐phosphonopentanoic acid (AP5) into the SON respectively increased or decreased the firing rate of OXT neurons and burst amplitude. However, it is critical to note that NMDA and AP5 did not affect the occurrence of bursts in non‐bursting OXT neurons. Therefore, Moos and colleagues reported that, although both NMDA and AMPA receptors are implicated in the enhancement of OXT neural activity and the stimulation of OXT release in vivo, NMDA receptors alone do not facilitate burst occurrence.

### 5.2 In vitro studies

Electrophysiological studies in male hypothalamic acute slices and organotypic slice cultures have further supported a stimulatory role for glutamate. Application of glutamate and its agonists kainate and quisqualate elicited excitatory postsynaptic potentials (EPSPs) in hypothalamic neurons,^44^, ^68^, ^69^, ^70^ whereas glutamate antagonists kynurenic acid and γ‐d‐glutamylglycine attenuated spontaneous and evoked EPSPs.^70^, ^71^ Application of the non‐NMDA receptor antagonist CNQX consistently blocked spontaneous and evoked EPSPs in several studies.^43^, ^48^, ^70^, ^72^ The reported effects of NMDA receptors varied across studies employing different recording techniques in distinct in vitro preparations; however, most observed a modest effect of NMDA and its antagonist AP5 on postsynaptic activity compared to CNQX.^44^, ^68^, ^69^, ^70^, ^71^, ^72^ Metabotropic glutamate receptors have also been shown to contribute to hypothalamic neurosecretory cell activity, through both presynaptic modulation of glutamate release^73^ and postsynaptic suppression of potassium currents.^74^ Therefore, it is likely that metabotropic and ionotropic receptor subtypes are all implicated in maintaining excitatory neurotransmission in hypothalamic OXT neurons. However, their particular roles and degree of influence may vary based on the physiological circumstances under which they are recruited.

Although outside the scope of this review, it should be noted that other key neurotransmitters, such as GABA,^75^, ^76^, ^77^, ^78^, ^79^ noradrenaline,^61^, ^80^, ^81^ dopamine^82^, ^83^, ^84^ and acetylcholine,^85^, ^86^, ^87^ have been shown to modulate magnocellular neuron activity/release and likely work in conjunction with glutamate and OXT to facilitate activity in a circuit‐specific, context‐dependent manner. More thorough reviews on the effects of these neurotransmitters in the hypothalamus are available.^88^, ^89^, ^90^, ^91^, ^92^, ^93^

## 6 GLUTAMATERGIC CIRCUITS IN THE HYPOTHALAMIC PVN AND SON

Evidence for glutamatergic synaptic innervation of hypothalamic magnocellular neurosecretory cells has been supported by both structural and functional studies. Several ultrastructural immunocytochemistry studies conducted in male and female rodents have revealed that presynaptic axon terminals with glutamatergic immunoreactivity make contact with dendrites and cell bodies of AVP and OXT neurons. Approximately 20% of all presynaptic axon terminals on hypothalamic neurosecretory cells are glutamatergic, with many forming “shared” synapses in which terminals make contact with two or more postsynaptic cell bodies/dendrites.^69^, ^70^, ^79^, ^94^, ^95^, ^96^, ^97^ Furthermore, vesicular glutamate transporters (VGLUTs), specifically VGLUT‐2 and VGLUT‐3, are also detected within hypothalamic neurons.^98^, ^99^, ^100^ Functional evidence for glutamatergic circuits in magnocellular hypothalamic nuclei was established through in vitro recordings conducted in slices of adult male rat hypothalamus.^70^, ^71^, ^72^, ^80^, ^101^, ^102^, ^103^ These recordings demonstrated that extracellular electrical stimulation elicited EPSPs/excitatory postsynaptic currents (EPSCs) in magnocellular neurons that were characterized by both synchronous and asynchronous glutamate release, which prolongs the excitatory synaptic response and increases the probability of firing multiple action potentials with each synaptic activation.^104^, ^105^ Together, these findings suggested that glutamatergic neurons input onto magnocellular neurosecretory cells in the PVN and SON, and that neurosecretory neurons in these nuclei are also glutamatergic themselves and may be capable of releasing both peptides and glutamate.

These discoveries coupled with observations concerning the effects of glutamate on OXT neuron activity brought the attention of the field to the potential role of local glutamatergic synaptic circuits in synchronizing the firing of OXT neurons. Activation of local circuits with glutamate microdrop application was shown to cause an increase in the frequency of EPSPs/EPSCs in some neurons,^102^ and EPSPs induced by noradrenaline were shown to be mediated by glutamate, given that bath application of both 6,7‐dinitroquinoxaline‐2,3‐dione (DNQX) and AP5 blocked these excitatory responses.^80^ The noradrenaline‐induced increase in EPSPs was retained in brain slices in which tissue surrounding the PVN had been trimmed away, suggesting that the presynaptic noradrenaline‐sensitive glutamatergic circuits are intrinsic to the PVN. Notably, EPSP/EPSCs in the PVN could also be generated by microdrop stimulation of the ipsilateral SON or contralateral PVN, which were also blocked by DNQX and AP5.^101^ Together, these ex vivo findings suggested the existence of local glutamatergic circuits and showed that synchronization between neurons in the PVN and SON could be achieved, at least in principal, by local glutamatergic circuits.

**FIGURE 1:**
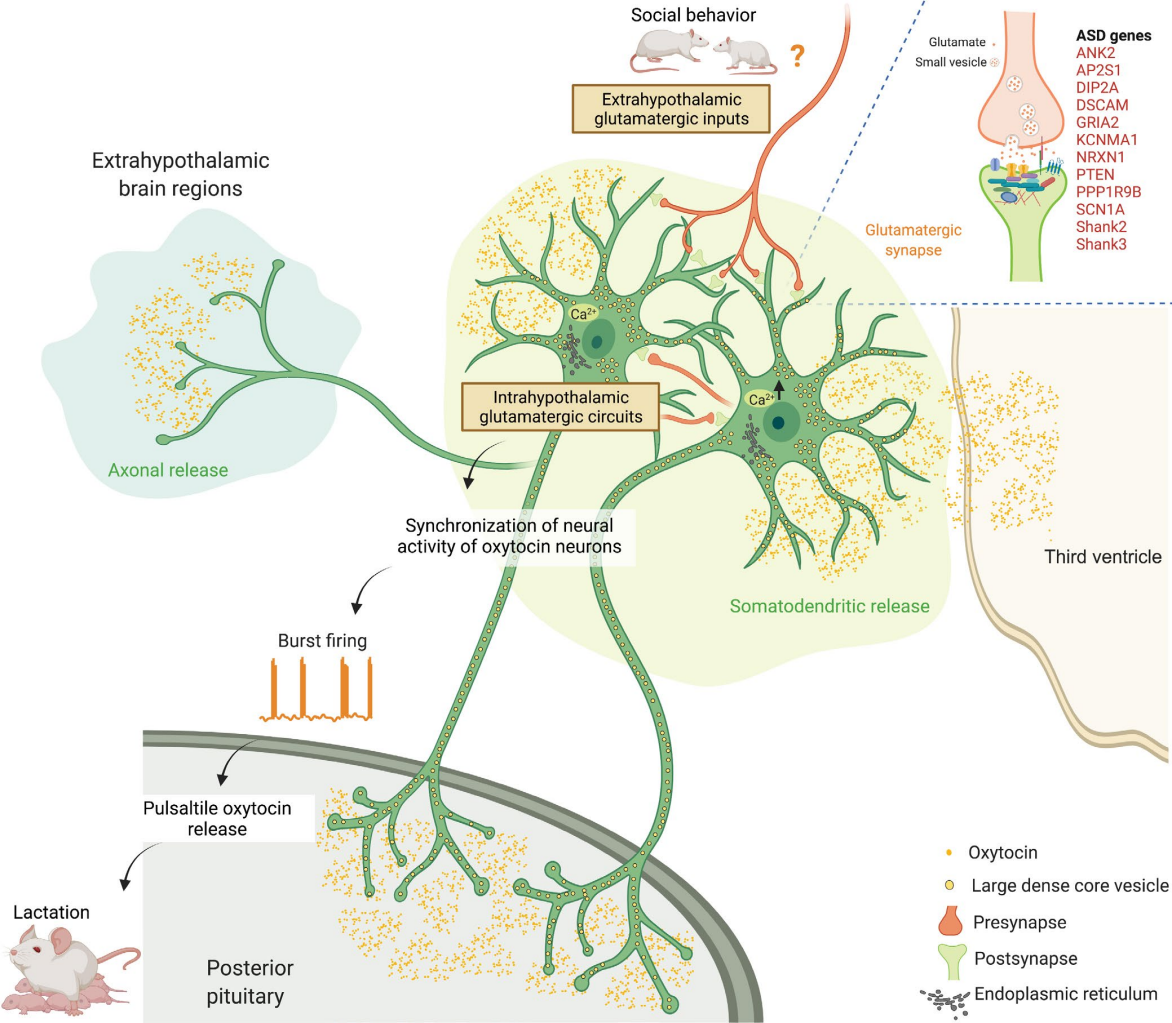
Hypothalamic glutamatergic circuits synchronize the activity of oxytocin neurons, leading to burst firing during lactation, which then leads to pulsatile release of oxytocin into the bloodstream to stimulate milk ejection. Extrahypothalamic glutamatergic inputs innervate oxytocin neurons, yet their role in modulating oxytocin neural activity and social behavior is not fully understood and could be of relevance for autism spectrum disorder (ASD), given the recent discoveries of several autism‐risk genes (in red) encoding for glutamatergic synaptic components, including adhesion molecules, scaffolding proteins and glutamatergic receptor subunits

Direct evidence for synchronous synaptic inputs to OXT neurons from local glutamate circuits came from electrophysiological recordings in organotypic slice cultures of the hypothalamus. These slice cultures retain a certain number of cells intrinsic to the slice section and maintain the gross synaptic organization of the original tissue after several weeks in culture.^44^ Using this approach, Poulain and colleagues were able to record synchronized bursts of glutamatergic EPSPs in identified OXT neurons that drove synchronous high frequency bursts of action potentials among pairs of OXT neurons.^43^, ^48^, ^106^ This, therefore, provided direct evidence for synchronized bursting in OXT neurons coordinated in part by the activation of local hypothalamic glutamatergic circuits (Figure 1). Although excitatory synaptic connections generated by the slice culture conditions which are not normally present in native hypothalamic tissue may have contributed to synchronization of the OXT bursts in these studies, this finding nevertheless indicated that intrinsic glutamate circuits were present at the time the hypothalamus was isolated. These same regenerative properties of the slice culture preparation may have also contributed to synchronized synaptic inputs and action potentials in this preparation that have not been observed in acute slices or in vivo.

## 7 EXTRAHYPOTHALAMIC GLUTAMATERGIC INPUTS TO THE PVN AND SON

Although studies conducted in vitro have greatly informed our understanding of the patterns of OXT neural activity and the release of OXT peptide in response to glutamate, the question of how sensory stimuli in the environment translate to neural signals to affect the pattern of hypothalamic OXT neurons remains of interest. Some insight on this matter emerged from studies in female rats where somatosensory information from the uterus, during parturition, or the nipples, during suckling, has been shown to trigger OXT release via the activity of A2 adrenergic afferents from the nucleus tractus solitarius of the brainstem to the PVN and SON.^107^, ^108^, ^109^, ^110^, ^111^, ^112^ A2 adrenergic afferents release noradrenaline, which acts on local glutamatergic circuits within the PVN and SON, leading to changes in the firing pattern of OXT neurons and to the release of the OXT peptide.^75^, ^80^, ^113^, ^114^ Furthermore, several in vitro experiments have shown that noradrenaline depolarizes magnocellular neurons by direct ionic mechanisms.^114^, ^115^ Finally, noradrenergic neurons have also been shown to produce and release glutamate, suggesting that glutamate and noradrenaline are likely to be co‐released from hypothalamic neurons.^116^, ^117^, ^118^, ^119^ More work needs to be done to achieve a better understanding of how external sensory stimulation, such as baby vocalizations, suckling, social interaction and social transmission of maternal behavior, which was recently shown to be mediated by OXT,^120^ lead to activation of OXT neurons and to the release of OXT peptide, as well as which inputs on OXT neurons play a role in this process. To identify glutamatergic/aspartergic inputs to the PVN and SON, Csáki and colleagues used the retrograde tracer [^3^H]D‐aspartate coupled with autoradiography in male rats.^121^, ^122^ They found that both the PVN and SON receive excitatory inputs from the thalamus, septum, bed nucleus of the stria terminalis and amygdala. They also found that cell bodies within several hypothalamic nuclei, including the PVN and SON, were labelled with [^3^H]d‐aspartate, indicating that these nuclei are interconnected by glutamatergic neurons. It is important to note that, although the approach applied in these studies provided important information on the origins of excitatory inputs to the hypothalamus, it lacked specificity because it is not selective for inputs to OXT neurons; therefore, the definite identity of these inputs could not be accurately determined. Recently, Tang and colleagues applied a retrograde tracing approach in females which allowed for the detection of afferents that specifically innervate OXT neurons. Using a viral system based on a retrograde trans‐synaptic EnvA‐pseudotyped G deletion‐mutant rabies virus in combination with a helper adeno‐associated virus expressed under the OXT promoter,^123^ they found that OXT neurons of the PVN receive afferents from several brain structures including the septum, medial preoptic area, insula, habenula, paraventricular nucleus of the thalamus, amygdala and substantia nigra. Although the identity of these inputs was not determined in their study, these findings in conjunction with those from Csáki and colleagues^121^, ^122^ suggest that some of these afferents may be glutamatergic, and the question remains as to whether these glutamatergic inputs from extrahypothalamic brain regions play a role in the regulation of OXT neural firing and, if so, what behavioral or physiological mechanisms engage these circuits (Figure 1).

## 8 PHYSIOLOGICAL RELEVANCE OF GLUTAMATERGIC CIRCUITS

Our enhanced understanding of the role that glutamate plays in regulating OXT neural activity and the presumption, based on independent observations that OXT neurons are innervated by glutamatergic afferents, raises new questions about the physiological relevance of the hypothalamic glutamate‐OXT circuits. It has been demonstrated, based on several in vivo studies, that intrahypothalamic glutamate may contribute to OXT neuron activity and release during lactation in vivo^65^, ^66^, ^67^ and synchronized burst firing in vitro,^43^, ^48^, ^80^, ^101^, ^102^, ^103^ but interpretation of these data together is complicated by key differences in the model systems utilized in these studies. Originally, it was assumed that OXT cell burst activity is exclusive to females during parturition and lactation, given that evidence of synaptic remodeling has been reported in hypothalamic OXT neurons under these physiological conditions.^89^, ^91^, ^124^, ^125^ However, in vitro studies examining this mechanism have primarily used hypothalamic tissue obtained from adult males^46^, ^68^, ^72^, ^80^, ^101^, ^102^, ^103^ or neonates^43^, ^44^, ^48^ and it is unknown whether this activity occurs naturally in vivo in these populations and, if so, under what circumstances. Glutamate has been implicated in stimulating OXT release to regulate cardiovascular activity^62^, ^63^ and during dehydration.^126^ Although the function of glutamate‐OXT circuits in the context of social behaviors remains unknown, both glutamate and OXT have been separately implicated in neurodevelopmental disorders characterized by deficits in social behavior.^127^, ^128^, ^129^, ^130^

## 9 IMPLICATION OF GLUTAMATERGIC CIRCUITS AND OXYTOCIN IN NEURODEVELOPMENTAL DISORDERS

Glutamatergic signaling plays a pivotal role in early brain development by regulating the proliferation and differentiation of neural progenitor cells,^131^, ^132^ neuronal migration^133^, ^134^ and synaptic plasticity.^135^, ^136^, ^137^ These processes are critical for the proper formation of brain circuits from pre‐birth into adolescence.^138^, ^139^ During this period, symptoms indicative of neurodevelopmental disorders (NDDs), such as intellectual disability and ASD, may also emerge. Research suggests that the timing of these critical periods and the emergence of NDDs do not co‐occur by chance, and that glutamatergic signaling plays a significant role in the pathophysiology related to these disorders,^127^, ^140^, ^141^ as well as other co‐morbid disorders such as epilepsy^142^, ^143^ and schizophrenia.^144^, ^145^ A predominant theory in the field of NDDs is that an imbalance in excitatory/inhibitory synaptic transmission caused by deficits in synapse development, signaling or plasticity may underlie cognitive and social impairments associated with NDDs.^146^, ^147^, ^148^ This theory is in line with the fact that many genes encoding for glutamatergic receptors, synaptic cell adhesion molecules and synaptic scaffolding proteins have been implicated in conferring risk for NDDs (Figure 1).^127^, ^128^, ^141^, ^149^, ^150^, ^151^ To investigate the effects of gene mutations such as these on the brain, several rodent models have been generated and shown to exhibit deficits in sensory and motor function, learning and memory and communication/social behavior.^152^, ^153^, ^154^, ^155^, ^156^, ^157^ These behavioral deficits were accompanied by alterations in glutamatergic synapse structure and function, which have been mostly assessed in the striatum^152^, ^158^, ^159^, ^160^, ^161^ and the hippocampus.^162^, ^163^, ^164^ Few pre‐clinical studies in animal models have assessed the impact of mutations in ASD high‐risk genes on the hypothalamic OXT system, and those that have mainly focused on the overall number of OXT neurons in the PVN. They have also assessed the ability of the OXT peptide to improve behavioral deficits. For example, using a mouse model with a knockout of *Cntnap2*, a member of the neurexin family of cell adhesion molecules, Penagarikano and colleagues found that *Cntnap2*‐deficient male and female mice had lower levels of PVN‐OXT neurons and reduced levels of OXT in whole‐brain extracts compared to wild‐type littermates.^165^ Furthermore, they found that intranasal administration of OXT during development had lasting restorative effects on social behavior and the number of PVN‐OXT neurons in *Cntnap2*‐deficient mice and that chemogenetic activation of endogenous OXT neurons in the *Cntnap2*‐deficient mice similarly produced behavioral improvements. Lower levels of PVN‐OXT neurons, specifically in the medial region of the PVN, were also recently reported in a *Shank3*‐deficient male mice, which harbors a mutation in the *Shank3* gene that encodes for a postsynaptic density scaffolding protein of the glutamatergic synapse.^166^ Social deficits were reported in this mouse model and were rescued by intraperitoneal administration of an OXT receptor agonist, known to cross the blood–brain barrier.^167^ This study corroborated previous findings in *Shank3*‐deficient male rats in which deficits in social recognition memory, attention and synaptic plasticity were ameliorated by i.c.v. administration of OXT.^168^ However, whether perturbations in the OXT system are present in this rat model is yet to be determined. Similarly, a study in the glutamate receptor *Grin1*‐deficient mice demonstrated that subchronic intraperitoneal treatments with OXT in both males and females increased sociability in the three‐chamber test, yet the structure and function of hypothalamic OXT neurons were not assessed in these mice and it is unknown whether *Grin1* mutations affect the hypothalamic glutamate‐OXT system.^156^ This gap in knowledge emphasizes the need for more research that aims to unravel the impact of mutations in glutamatergic NDDs and ASD high‐risk genes on the functionality of the hypothalamic OXT system, specifically on the hypothalamic glutamate‐OXT circuits.

Currently, the field is well‐poised to address these questions given recent technological advancements.^169^ A key element that has been achieved is the ability to measure OXT neural activity in the hypothalamus during social behavior using fiber photometry^123^, ^170^ and two‐photon calcium imaging.^166^ Fiber photometry allows for high temporal resolution and free movement, yet low spatial resolution, whereas two‐photon imaging provides high spatial and temporal resolution with the disadvantage of limited mobility due to head fixation. Calcium imaging using the miniaturized fluorescence microscope (miniscope) is an additional technique that provides high spatial and temporal resolution at the same time as allowing rodents to freely move throughout their environment.^171^ Furthermore, the ongoing effort to develop OXT genetically‐encoded OXT fluorescent sensors will allow for more specific detection of OXT release. Critically, these methods can be used in combination with chemogenetics, optogenetics, transgenic rodents and viral vectors to address circuit‐specific research questions concerning the role of hypothalamic glutamate circuits and OXT neurons in physiological conditions, including behavior, and to investigate the implication of the OXT system in ASD and other NDDs.

## 10 CONCLUSIONS

The causality between OXT and ASD has been extensively discussed in the last two decades^172^, ^173^, ^174^, ^175^, ^176^, ^177^ and has been tested in several animal models for ASD and NDDs.^178^ This has led to increased interest in using the OXT peptide as a therapeutic to treat social behavior deficits in these disorders,^179^, ^180^, ^181^ with little attention being given to whether the OXT system is impacted by NDDs or ASD‐associated mutations. To date, there has been no genetic evidence that directly implicates the OXT or OXT receptor coding genes in NDDs. However, mutations in glutamatergic genes have been repeatedly identified in large‐scale genetic studies of NDDs,^150^, ^151^, ^182^ suggesting that glutamate‐OXT circuits could be perturbed in these disorders leading to dysregulation within the OXT system. The role of hypothalamic glutamate‐OXT circuits has been mostly studied in the context of lactation and parturition. Thus, we propose that future studies directed at dissecting the role of the intra and extrahypothalamic circuits in regulating the OXT system and modulating social behaviors will enhance our understanding of the mechanisms of function of the OXT system, and that investigating the impact of NDD‐associated mutation on the OXT system has the potential to uncover new pathophysiology that could be underlying social behavior deficits.

## CONFLICT OF INTERESTS

The authors declare that they have no conflicts of interest.

## AUTHOR CONTRIBUTIONS

**Amanda Leithead:** Conceptualization; Writing – original draft; Writing – review & editing. **Jeffrey G. Tasker:** Supervision; Writing ‐ original draft; Writing – review & editing. **Hala Harony‐Nicolas:** Conceptualization; Funding acquisition; Resources; Supervision; Writing – original draft; Writing – review & editing.

### PEER REVIEW

The peer review history for this article is available at https://publons.com/publon/10.1111/jne.13061.

## Data Availability

The present article comprises a review that does not report any novel data and so data sharing is not applicable.
